# Medical Content Searching, Retrieving, and Sharing Over the Internet: Lessons Learned From the mEducator Through a Scenario-Based Evaluation

**DOI:** 10.2196/jmir.3650

**Published:** 2015-10-09

**Authors:** Athos Antoniades, Iolie Nicolaidou, Dimitris Spachos, Jarkko Mylläri, Daniela Giordano, Eleni Dafli, Evangelia Mitsopoulou, Christos N Schizas, Constantinos Pattichis, Maria Nikolaidou, Panagiotis Bamidis

**Affiliations:** ^1^ Department of Computer Science University of Cyprus Nicosia Cyprus; ^2^ STREMBLE Ventures Ltd. Limassol Cyprus; ^3^ Department of Communication and Internet Studies Cyprus University of Technology Limassol Cyprus; ^4^ Lab of Medical Physics Medical School, Faculty of Health Sciences Aristotle University of Thessaloniki Thessaloniki Greece; ^5^ Department of Teacher Education University of Helsinki Helsinki Finland; ^6^ Dipartimento di Ingegneria Elettrica, Elettronica e Informatica Università di Catania Catania Italy; ^7^ e-Learning Unit (ELU), Division of Population Health Sciences & Education, St George’s University of London London United Kingdom

**Keywords:** searching and sharing of medical educational content, repurposing, metadata, evaluation

## Abstract

**Background:**

The mEducator Best Practice Network (BPN) implemented and extended standards and reference models in e-learning to develop innovative frameworks as well as solutions that enable specialized state-of-the-art medical educational content to be discovered, retrieved, shared, and re-purposed across European Institutions, targeting medical students, doctors, educators and health care professionals. Scenario-based evaluation for usability testing, complemented with data from online questionnaires and field notes of users’ performance, was designed and utilized for the evaluation of these solutions.

**Objective:**

The objective of this work is twofold: (1) to describe one instantiation of the mEducator BPN solutions (mEducator3.0 - “MEdical Education LINnked Arena” MELINA+) with a focus on the metadata schema used, as well as on other aspects of the system that pertain to usability and acceptance, and (2) to present evaluation results on the suitability of the proposed metadata schema for searching, retrieving, and sharing of medical content and with respect to the overall usability and acceptance of the system from the target users.

**Methods:**

A comprehensive evaluation methodology framework was developed and applied to four case studies, which were conducted in four different countries (ie, Greece, Cyprus, Bulgaria and Romania), with a total of 126 participants. In these case studies, scenarios referring to creating, sharing, and retrieving medical educational content using mEducator3.0 were used. The data were collected through two online questionnaires, consisting of 36 closed-ended questions and two open-ended questions that referred to mEducator 3.0 and through the use of field notes during scenario-based evaluations.

**Results:**

The main findings of the study showed that even though the informational needs of the mEducator target groups were addressed to a satisfactory extent and the metadata schema supported content creation, sharing, and retrieval from an end-user perspective, users faced difficulties in achieving a shared understanding of the meaning of some metadata fields and in correctly managing the intellectual property rights of repurposed content.

**Conclusions:**

The results of this evaluation impact researchers, medical professionals, and designers interested in using similar systems for educational content sharing in medical and other domains. Recommendations on how to improve the search, retrieval, identification, and obtaining of medical resources are provided, by addressing issues of content description metadata, content description procedures, and intellectual property rights for re-purposed content.

## Introduction

### The mEducator Best Practice Network and Solutions Proposed

Although there is an abundance of medical educational content available in individual academic institutions, it is not widely available or easy to discover and retrieve due to a lack of standardized content-sharing mechanisms. Medical education institutions often use a variety of Web-based Learning Content Management Systems (LCMSs) to support the teaching and learning process. They also use some of the available educational standards to describe the educational content in the LCMS. This would presumably allow for better managing of the content through the Web and increase its interoperability in different LCMSs and platforms, but instead these learning materials often remain confined within the individual institutions. Sharing educational material has been the focus of recent developments and practice. Web 2.0 has highlighted the importance of sharing, as well as social collaboration and participation, social networking, and crowd intelligence in the domain of health education, where medical doctors, medical students, practitioners, and others, can benefit from open access to information and sharing of ideas, questions, and opinions [1].

Despite numerous efforts and emphasis in the area of educational content development and its sharing through social media (eg, YouTube), there has been no prominent, clear, and standards-based solution for the seamless sharing of educational content in medicine, where seamless implies immediate awareness of any new educational content, accuracy of its classification regarding the topics and skills being addressed, and visibility of any existing adaptations of the content to suit different educational context (repurposing). For example, recent research has examined the use of a standardized medical thesaurus (SNOMED CT) in YouTube health video tags from preselected YouTube medical education channels and found that the average percentage of YouTube tags expressed using SNOMED CT terms was about 22% [2]. To fill this gap, the mEducator Best Practice Network (BPN) implemented standards and reference models in e-learning to develop innovative solutions that enable specialized state-of-the-art medical educational content to be discovered, retrieved, shared, and re-used across European Institutions, targeting medical students, doctors, educators, and health care professionals [3]. For this aim, mEducator also had to elaborate on pedagogical, technical, standardization, cultural, social, and legal issues.

mEducator has tackled the challenge of seamless sharing of educational content by fusing the social Web and the semantic Web concepts. Learning management systems and open educational repositories were united so that educators and learners could organize, repurpose (defined as convert for use in another format or educational context), re-use, and share medical educational resources. Different platforms have been created to enable the organization, repurposing, re-use, and sharing of medical educational resources. The mEducator ontology has played a pivotal role in this endeavor, as it has been designed to provide the various mEducator instances with a metadata schema that has well-defined semantics [1]. These instances, all using the same metadata schema, can be classified based on the specific underlying technologies: two solution frameworks that were developed for multitype content sharing and repurposing.

#### First Solution: mEducator2.0, Based on Web 2.0 Technologies (Specifically Mashups)

In mEducator2.0, a brokerage mechanism was created based on mashups and other Web 2.0 technologies, which allows medical educational content to be shared across LCMSs, thereby creating a loosely coupled network of LCMSs. A mashup is a Web application that uses content from more than one source to create a single new service displayed in a single graphical interface. Users can access mEducator educational material through the mEducator2.0 portal and their own systems using mashup technologies. mEducator users across multiple institutions may use the mashups for uploading, creating, and editing content metadata, as well as for the search and retrieval of content. Alternatively, for users without access to a specific LCMS, an independent platform has been created, which applies Web 2.0 techniques and facilitates user collaboration, allowing, at the same time, the creation of social networks for medical education and knowledge exchange [1].

#### Second Solution: mEducator3.0, Based on Semantic Web Technologies (Specifically Linked Data)

The mEducator3.0 solution is designed around a federated architecture based on a service-oriented application framework and the use of semantic technologies. The framework is fundamentally based on the Semantic Web Services-oriented e-learning architecture [4,5] and the emerging Linked Data and Linked Services paradigms [6]. This solution fundamentally exploits semantic representations of data and services to provide interoperability between e-learning repositories spread across the Web. In particular, descriptions of the content are based on machine-understandable metadata and vocabularies that also follow linked data principles [7,8].

There are four instantiations of the mEducator3.0 solution, which differ in the type of content management technologies (CMT) they are integrated with: (1) mEducator3.0-MILES+, based on semantic extensions of Moodle [9,10], (2) mEducator3.0-MELINA+, based on the Drupal content management system (CMS), (3) mEducator3.0-Linked Labyrinth+ [11], based on the Open Labyrinth platform for Virtual patients, and (4) mEducator3.0-Metamorphosis+, based on the social network, Elgg [12,13].

This paper focuses on the evaluation of MELINA+, with respect to the underlying metadata schema and the overall usability and user acceptance of the system. We chose to report on this solution because it was the most widely tested, in a number of different settings, in four different countries (ie, Greece, Cyprus, Bulgaria, and Romania), with a sufficient number of users. This study is original in implementing scenario-based assessment for evaluating content-sharing technologies in tandem with end-users, contents, and their interactions. The paper is structured as follows: the next section synthesizes the findings of a literature review on evaluating metadata in e-learning systems, which leads to the section where we define the term “metadata schema” and illustrate its importance in the context of mEducator. We then provide the rationale for using scenario-based evaluation to address the target users’ needs and conclude with the research goal of the study. The methodology section describes how scenario-based evaluation was conducted and provides information on the participants of the study and the instruments that were used. The results section presents the results of the evaluation of the metadata schema and the overall usability of the system, as well as how the latter was perceived and accepted by users. The discussion section provides recommendations and lessons learned from the evaluation and draws implications for the design of content sharing and repurposing systems for medical education.

### What We Know From Previous Research

#### Evaluating Metadata in E-Learning Systems: Research Background and Rationale

The evaluation framework of mEducator has drawn from the literature on the implementation and evaluation of metadata in other e-learning systems [14]. The term metadata is used to describe data that provide additional information about a certain item’s content, that is, data about data. A metadata schema is composed of a set of terms, a set of structural definitions of metadata instances, and a binding schema for implementation [15,16]. In the e-learning context, metadata, by providing descriptive information about resources and learning objects, facilitate retrieval and re-use in various instructional contexts and have been researched extensively [17]. One such example is the Adaptive Hypermedia Knowledge Management E-Learning Platform, which used metadata to satisfy requirements such as reusability and interoperability and provided tools to support teachers in the evaluation, import, and retrieval of high-quality educational resources. Rego et al [17] focused on the evaluation of the quality of learning objects and developed a tool that involved the use of an intelligent agent using data mining techniques for the analysis of the metadata contained in the learning object.

Researchers, who concentrated their efforts on designing a system where users could most effectively find the items they searched for, agreed on two crucial factors: (1) the effective use of metadata [18] and (2) a user-centered approach [19-21]. Morales-Salcedo et al [19] in their U-campus (Ubiquitous campus) project, provided users with means to access and control all available resources in a uniform fashion from a single vantage point. Gkatzidou et al [20] adopted a user-centered approach in their effort to create reusable, accessible, and adaptable learning objects, by focusing attention to user profiles. In their case, metadata were used to describe not only the learning object but also the learner’s profile. Specifically, the description of the user’s immediate needs and preferences was matched with a description of the components of a resource or service to provide an accessible relationship between the learner and the resource and enable the delivery of learning content that has been adapted to suit the needs of the individual user. However, they did not provide a way for evaluating their approach.

To reduce the subjectivity in the evaluation of the quality of metadata, some researchers [22] borrowed the evaluation framework proposed by others [23] and provided methodological guidelines that are useful for the evaluation of metadata in other types of systems. This framework summarized the quality of the metadata instance in seven measurable parameters: (1) completeness, (2) accuracy, (3) provenance, (4) conformance to expectation, (5) logical consistency and coherence, (6) timeliness, and (7) accessibility. As explained in [22]:

In a complete metadata record, the learning object is described using all the fields that are relevant to describe it. In an accurate metadata record, the data contained in the fields correspond to the object that is being described. The provenance parameter reflects the degree of trust that you have in the creator of the metadata record. Conformance to expectations measures how well the data contained in the record let you gain knowledge about the learning object without actually seeing the object. Logical consistency and coherence reflects two measures: The consistency measures if the values chosen for different fields in the record agree between them. Coherence measures if all the fields talk about the same object. Timeliness measures how up-to-date the metadata record is compared with changes in the object. Accessibility measures how well you are able to understand the content of the metadata record (p. 6).

Adopting a user-centered approach and recognizing both the limitations of having experts create metadata and the limitations of automatically generating them, Zens and Baumgartner [21], in their effort to allow users to find resources that fit their needs, suggested social tagging of metadata in the context of education as a third way of creating metadata. The pedagogical potential of social bookmarking was also critically presented by Dias et al [24]. The system by Zens and Baumgartner [21] was called MELT (A Metadata Ecology for Learning and Teaching), and it represents a content enrichment project that bridged 17 public and private sector content partners with the goal of promoting the exchange of learning resources across Europe. MELT used an existing brokerage system that supported federated searching across a network of linked content repositories. MELT pursued a multilayer metadata enrichment approach that included expert indexing, automatic metadata generation, and social tagging. Social tagging allowed users to add tags to given objects and thus reflected the view of multiple users. It resulted in many accumulative metadata records related to a given resource. Social tagging results in folksonomies. Folksonomies are user-generated taxonomies that facilitate the sharing of content within a social network of users and potentially promote an efficient discovery of learning resources that meet the user’s needs. The potential of taxonomies for learning objects in the medical domain has been explored by mEducator in [25]. A similarity between MELT and mEducator is that mEducator also allows users to add open-ended metadata fields when adding content in the platform [25], when the existing metadata do not cover a specific aspect they consider important. For the evaluation of the effectiveness of MELT, seven success indicators were implemented, some of which are applicable in the context of mEducator, as well. Those success indicators are the following: (1) effectiveness and efficiency of the search and retrieval process, (2) utility of metadata enriched by experts for finding relevant content, (3) utility of folksonomies for finding relevant content, (4) effectiveness of automatically generated metadata regarding discovery of content, (5) user satisfaction, (6) use of the retrieved content, and (7) use of content across languages and across countries.

#### Evaluating Metadata in mEducator

The term “metadata schema” is used in mEducator to describe medical educational resources of various types in a standardized, machine processable format in order to enable the medical educational resources to be shared, exchanged, searched, and retrieved across academic institutions. One key challenge was that the completion of metadata is not performed by professional indexers, as was the case in previous research [26], but is largely delegated to end-users and content providers. This is challenging because, in general, it is difficult for the end-users to achieve a shared understanding of the meaning of the metadata fields, as demonstrated in studies of digital libraries [27] and in some preliminary studies carried out within mEducator [28].

**Figure 1:**
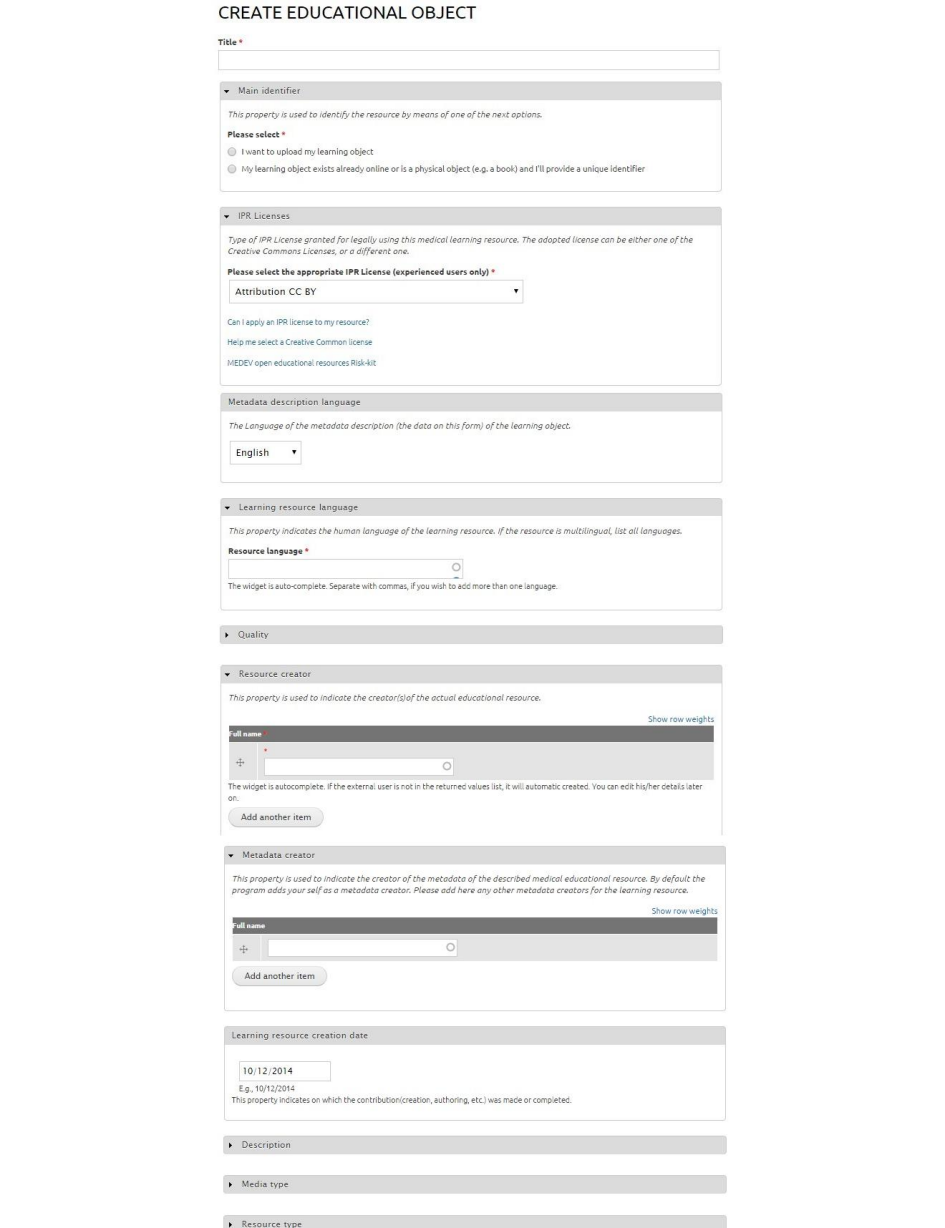
The metadata filling interface in meducator MELINA+.

The approach followed by mEducator in the design of the metadata schema was based on an attempt to balance competing requirements. Specifically, the number of required fields, including ones deemed essential to the mEducator content-sharing model, that is, specification of the intellectual property rights (IPR) for the shared content and the description of the content modification carried out during repurposing extensions was minimized, while the opportunity for richer descriptions of the resource was still provided in the system. Controlled vocabularies/taxonomies were introduced to facilitate classification of the educational resource. A set of additional optional fields were proposed for a more comprehensive description of the educational/pedagogical aspects of the content. The metadata-filling interface in mEducator MELINA+ is shown in Figure 1. After providing a title, a user had to specify fields, such as IPR licenses, metadata description language, learning resource language, quality, resource creator, metadata creator, learning resource creation date, description, media type, resource type, and discipline (Figure 1).

The evaluation of the mEducator3.0 solution MELINA+ was based on an evaluation framework specifically developed for assessing the effectiveness of all the platforms that were implemented through the mEducator BPN and any future platforms that attempt to address the same issues. An a priori analysis of the goals and nature of the mEducator solutions pointed to five important pillars that need to be addressed in an evaluation model [29]: (1) IPR of content (refers to license types and mechanisms of content protection), (2) repurposing (refers to the tracking of content genealogy and content re-use activities), (3) accessibility of metadata schema, (4) content evaluation (system generated analyses, user review, peer review process), and (5) content sharing. At the same time, three dimensions are transversal and span those pillars: (1) human-computer interaction (HCI) (overall accessibility, usability, consistency of the proposed solutions), (2) technological issues (requirements for content providers, handling of content updates, system performance, and maintenance requirements), and (3) sustainability (incentives for content providers/ consumers/ reviewers and financial sustainability).

mEducator attempted to take the context of the target users’ activities into account in the evaluation effort. Accordingly, the targeted user groups serve as the point of departure for designing and executing user testing. Evaluation is based on scenarios typical for each user group’s context of using Web services (scenario-based evaluation). These activity scenarios also take into account the dominant or typical content types for each user group and context. The decision to use activity scenarios is supported by previous research that showed that good assessment scenarios are particularly revealing and valuable because they ask learners to make decisions by applying their understanding of the system [30].

### Scenario-Based Evaluation: Research Background and Rationale

Several research attempts have focused on developing a scenario-based evaluation model in diverse disciplines, such as the online assessment of students’ problem solving skills [30], educational assessment [31], service-oriented architectures [32], and software engineering [33], to name a few. In medical education in particular, scenario-based assessment is being used extensively for the assessment of clinical skills [34] or for training [35] or even for exploring design requirements for repurposing medical cases [36].

In the context of educational assessment, Mayotte [31] attempted to evaluate the effectiveness of an interactive scenario-based assessment system to address the limitations of traditional assessment methods, allowing students to troubleshoot complex scenarios, ask questions, and make diagnoses through an interactive Web interface. His research showed that scenario-based assessment placed greater emphasis on learners’ problem solving, critical thinking, and reasoning skills. In the context of educational assessment within medical education, Nestel et al collected performance data [34] when third-year medical students worked through scenarios undertaking defined tasks for the assessment of their procedural skills in contextualized (specific objective-driven) tasks. In the latter research [34], it was found that scenario-based evaluation was valued for the opportunity to practice patient-centered care in a simulated setting that integrated technical, communication, and other professional skills. These researchers concluded that scenario-based assessment reflected real-world issues of patient-centered care [34]. In the context of scenario-based training in medical education, similar findings were reported by [35] who found that scenario-based virtual world team training of cardiopulmonary resuscitation (CPR) was feasible and showed promising results for the training of medical students in multiperson CPR. Moreover, the value of scenario-based think aloud protocols in the area of symptom interpretation, online diagnosis, and HCI was demonstrated by [37] in their work with adults aged over 50 years.

To the best of our knowledge, no research studies on scenario-based assessment for evaluating content-sharing technologies in tandem with end-users, contents, and their interactions exist in the literature [38].

### Research Goal

The focus of the evaluation methodology was to define the extent to which the informational needs of the mEducator target users were covered and if the schema supported content searching, retrieval, and sharing from an end-user perspective. For example, the construct “metadata accuracy” was operationalized to address the users’ notion of whether the metadata are understandable, whether and how they cover the informational needs of the mEducator target group, and how they function to retrieve relevant content. The term “accuracy” is therefore referred to in this study in its broader sense of general adequacy for the target users’ needs, rather than in the more technical acceptance of the term, often used in the literature, to indicate whether in any digital collection the metadata are filled with accurate/precise information with respect to the digital object they refer to.

The research goals of our study were to assess (1) how the metadata schema developed by mEducator addresses the informational needs of end-users in the process of medical content searching, (2) how the metadata schema developed by mEducator supports the process of content sharing, (3) the overall usability of the schema as implemented in the mEducator3.0 MELINA+ system, and (4) the overall user acceptance of this particular mEducator system.

The evaluation relates to the medical domain in three main ways: (1) some fields of the metadata schema refer to the medical/health domain and thus are very specific, (2) the testing scenarios used in the evaluations are largely dominated by medical/health-related cases in which the aforementioned medical-domain–related metadata are involved, and (3) the whole evaluation context was medical, in the sense that participants were either medical students (undergraduate or post-graduate) or health professionals. So in this study, (1) medical-domain specific metadata were defined and used, (2) medical-domain–related scenarios were constructed and used, and (3) all participants were from the medical domain.

## Methods

### Scenario-Based Evaluation Context

**Figure 2:**
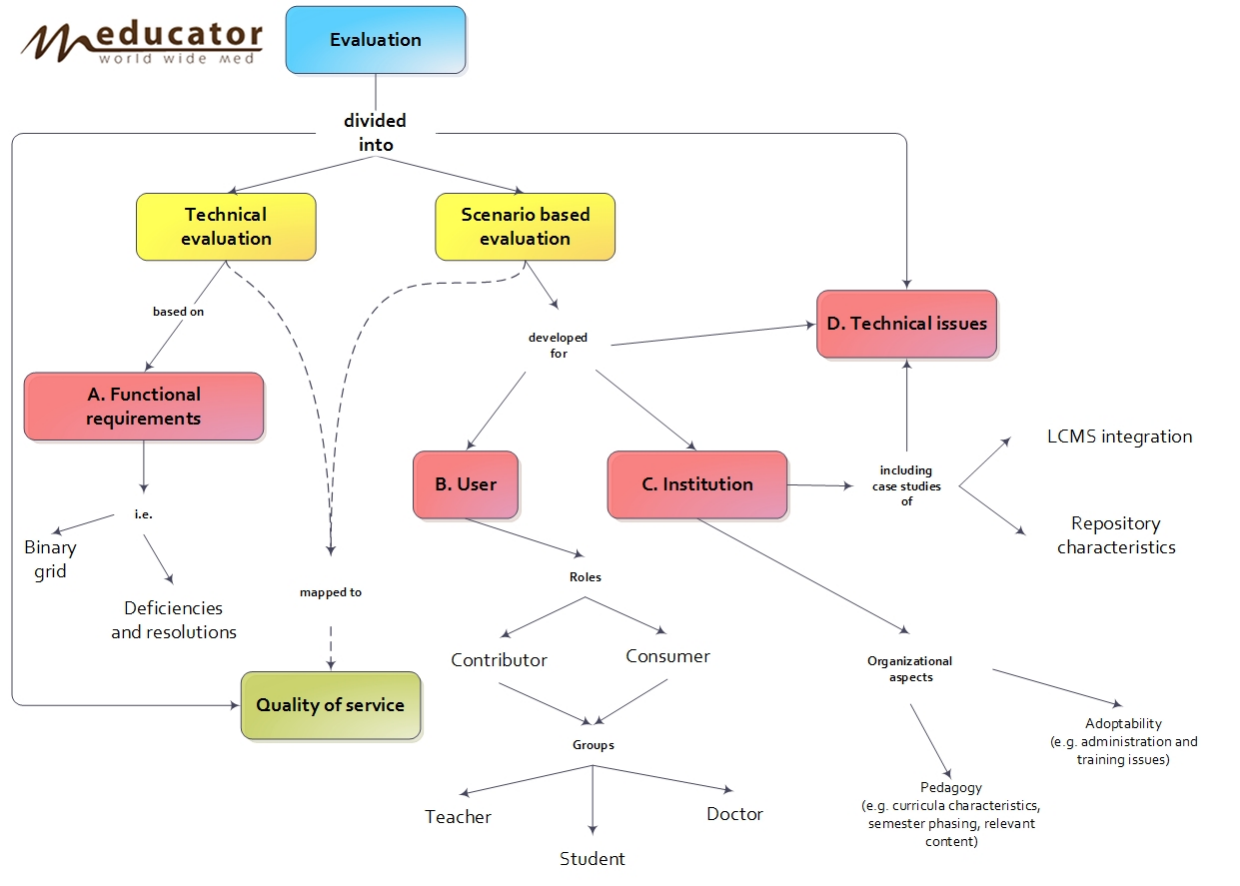
Overview of the evaluation framework developed for the mEducator system, dividing evaluation into technical evaluation and scenario-based evaluation.

A number of assessment methods, including scenario-based assessment for usability testing complemented with data from online questionnaires and field notes, were designed and used to evaluate the different instantiations of the mEducator solutions. Overall, the evaluation effort in mEducator addressed two main levels: (1) the system perspective, that is, technical evaluation, based on functional requirements, and (2) the usage perspective, that is, scenario-based evaluation. Both were carried out with a focus on quality of service and user experience. The scenario-based evaluation across user groups was decided with a special emphasis on the user testing stage. A framework for integrated scenario-based and quality of service evaluation was developed (Figure 2). The overall evaluation framework for the mEducator system was divided into technical evaluation, which was based on functional requirements, and scenario-based evaluation, which is the focus of this study.

As Figure 2 shows, “scenario-based evaluation” was developed for the individual “users” and “institutions” but also addressed technical issues, always taking into consideration the target users’ (“teachers”, “students”, and “doctors”) roles as “contributors” or “consumers” of medical educational material in the mEducator system (Figure 2). Case studies examining issues of pedagogy and adoptability in individual institutions, as well as technical issues of LCMS integration and repository characteristics, which are shown in Figure 2, are beyond the scope of this paper. It is important to note that different data sources and methods were used for the different levels of the evaluation. For example, in the scope of the overall study, interviews, observational case studies, screenshot capturing, and automated tracking of activity were implemented, primarily in the initial stage of the project to inform and evaluate the first prototypes of the mEducator system.

Scenario-based evaluation allowed the examination of key areas of the metadata schema, such as the following: (1) capability to embrace all types of medical learning resources, (2) capability to account for content repurposing, and (3) handling copyright licenses, especially of the parent resource during repurposing.

Evaluation workshops, referred to as case studies in this paper, were organized and run at various mEducator partners’ sites. This paper focuses on the analysis of four such case studies, through which the mEducator evaluation framework was applied. These case studies were conducted between August 31, 2011, and April 7, 2012, and they focused on one mEducator instantiation, MELINA+, which provided options to users to explore a large collection of educational objects, create their own educational object including a full metadata description, and collaborate with other members of the community.

**Figure 3:**
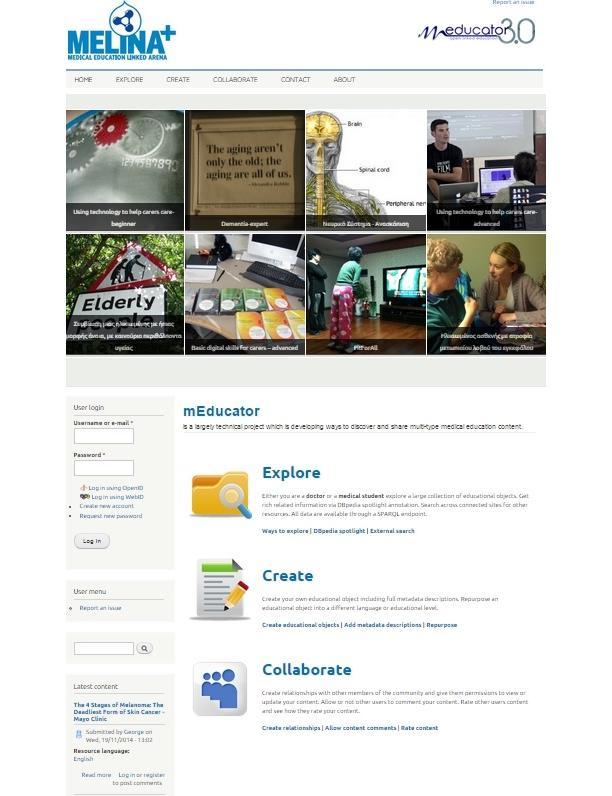
Screenshot of the interface of mEducator3.0 MELINA+ instantiation.

More specifically, MELINA+, which stands for MEdical Education LINnked Arena, is a CMS for medical educational resources, based on Drupal 7, an open source content management system. MELINA+ has been developed to allow resources to be created, uploaded, described, shared, and searched over the semantic Web in different ways. It exploits SPARQL queries using the Drupal SPARQL endpoint functionality. Multiple endpoints (internal and external) could be added in SPARQL registry and queried. It supports the creation and description of learning resources, user registration/authentication, advanced search capabilities, a commenting/rating/bookmarking system, blogs, and posts. Its advanced features include core RDF support, embedded SPARQL endpoint, DBpedia spotlight annotation, social learning collaboration, quality process control for learning resources, and single sign-on via WEBID module. Figure 3 shows the interface of MELINA+, specifically users’ options to “explore”, “create”, and “collaborate”.

Evaluation sessions started with a demonstration of the mEducator system conducted by a facilitator. It is important to clarify that scenario-based evaluation involves a facilitator that guides participants, especially when the latter are not familiar with a particular system, and as such it is a method typically applied in structured training and evaluation sessions. In the four case studies described in this paper, a facilitator and 2 research assistants were present to guide participants and also to take field notes based on their observations concerning the points where the target users faced difficulties or expressed doubts. Research assistants were instructed to take note of explicit requests for clarification when these were made by participants and also to take notes of the context in which the users seemed to be stuck or puzzled while performing each scenario.

In the context of scenario-based evaluation, participants typically created a learning resource, for example a presentation outlining the aims and results of a medical paper, complemented with educational information, such as educational objectives, target audience, and expected learning outcomes.

Participants had access to a number of different types of learning content in mEducator, in different languages, including text, multimedia slide presentations, images, videos, interactive learning environments, wikis, sketch graphical annotations, virtual patients, websites, animations, audio files, 3D models, and eBooks, all in the medical domain. Each case study included three scenarios, as described below.

#### Scenario 1: Creating an Educational Medical Resource and Its Metadata

The first scenario referred to the *creation* of an educational resource in MELINA+ (Figure 3, “Create”). Participants created a new account, logged into the system, uploaded a learning resource, entered and saved the appropriate metadata for the resource, and visualized the entered metadata in the system. Before they were in a position to evaluate MELINA+, they had to create an educational resource in mEducator3.0 MELINA+ and familiarize themselves with the medical educational resource creation processes. The role of the facilitator in Scenario 1 was to (1) explain the basic functionalities involved in medical educational resource creation, (2) explain the notion of metadata, especially those describing the resource in medical terms, and (3) help users appreciate the notion of the IPR metadata used for describing medical educational resources when it comes to sharing and repurposing medical content, even for educational uses.

#### Scenario 2: Searching for a Medical Educational Resource

The second scenario referred to the process of *searching* for a resource in MELINA+ (Figure 3, “Explore”). Before they were in a position to evaluate MELINA+, participants were expected to specify the search attributes, perform the search, and analyze the obtained results. The aim of Scenario 2 was to familiarize users with the searching of resources in mEducator3.0 MELINA+. The role of the facilitator in Scenario 2 was to (1) demonstrate to medical users how to search for educational resources in comparison to simple search engines, (2) explain the role of search attributes, and (3) help medical users appreciate notions of metadata used for describing the medical resource (and its subsequent sharing with other medical users).

#### Scenario 3: Repurposing and Specifying Intellectual Property Rights Attributes for Sharing Medical Resources

The third scenario referred to *repurposing* a resource and treating the IPR aspects of it (Figure 3, “Collaborate”). It is important to note that, by the third scenario, participants have already encountered the notion of IPR, as clearing IPR is compulsory in creating and sharing a resource. Before they were in a position to evaluate MELINA+, participants were expected to understand the notion of IPR and creative commons, specify the IPR metadata and attributes for their own resources, perform some kind of repurposing, correctly fill in repurposed resources, and analyze the obtained results. The role of the facilitator in Scenario 3 was to (1) help medical users familiarize themselves with the aspects of medical resource repurposing in mEducator3.0 MELINA+, (2) help medical users familiarize themselves with the notion of utilizing/exploiting metadata to link resources in an hierarchic way suitable for sharing in the medical education domain, and (3) explain to medical users the basic notion of repurposing in practice and its importance in medical education.

It is important to note that too few of the participants were able to complete the third scenario that referred to repurposing with regard to sharing within the allocated time. Therefore, this study focused primarily on creating, searching, and retrieving medical educational resources. Another important note is that the concept of repurposing is mostly of interest to professors and medical professionals, rather than to medical students, who constituted the majority of the participants in this study.

At the end of their interaction with MELINA+, participants completed two evaluation instruments, which are described in detail in the next section. Participants also completed two open-ended questions, which focused on the perceived system’s strengths and weaknesses.

### Participants

Overall, there were 126 respondents who evaluated the mEducator3.0 instantiation MELINA+. The majority of these respondents (77.8%) were undergraduate students, followed by 8.7% of graduate students, 6.3% of postgraduates, 4% of medical professionals, and 3.2% of professors.

### Instruments

Two main instruments were used as data sources for the mEducator evaluation framework: (1) the “System Usability Scale (SUS) questionnaire” and (2) the “Questionnaire on the metadata and search process”. The first instrument was primarily used to assess the overall usability of the system in absolute terms. The second instrument was primarily used to document (1) the extent to which the metadata schema developed by mEducator addresses the end-users’ informational needs in the process of medical content searching and (2) the extent to which the metadata schema developed by mEducator supports the process of content sharing. Both instruments were completed online in the form of an online anonymous survey.

Moreover, another source of data referred to the facilitator’s and research assistants’ field notes during the MELINA+ evaluation sessions, which documented cases where users expressed doubts or faced difficulties during the execution of the scenarios. These field notes were primarily used as a complementary source of data for triangulation purposes and also to help derive recommendations for the improvement of the system, based on the users’ input. The analysis of the first two data sources, the two instruments, includes (1) quantitative results from the completion of online surveys, and (2) qualitative results of open-ended questions in these surveys.

#### The System Usability Scale Questionnaire

The System Usability Scale (SUS) questionnaire (see Multimedia Appendix 1 for full instrument) provides a well-known, widely used, and standardized quantifiable measure for the usability of systems, which is provided in the form of an absolute scale that allows comparisons between systems. Selecting a standardized method that would allow the evaluation and comparison of the different instantiations of mEducator was a critical factor that had to be considered during the mEducator project.

The SUS questionnaire consists of 10 questions alternating in positive and negative phrasing to remove any bias arising from the phrasing of questions [39]. Each question is answered on a Likert scale from 1 (strongly disagree) to 5 (strongly agree). An advantage of the SUS questionnaire is that it produces a standardized score ranging from 0-100 that can be used to compare the usability of systems directly [40].

With regard to how the results of the SUS are analyzed, this instrument yields a single number representing a composite measure of the overall usability of the system being studied. Scores for individual items are not meaningful on their own. The procedure followed for scoring the SUS questionnaire and the results was the following: for odd questions, 1 was subtracted from the user response, and for even-numbered questions, the user response was subtracted from 5. This scales all values from 0-4 (with 4 being the most positive response). The converted responses were added up for each user and the total was multiplied by 2.5. This converted the range of possible values from 0-100 instead of 0-40.

#### Questionnaire on the Metadata and Search Process

The second instrument consisted of 16 questions that focused on the metadata and search process evaluation (see Multimedia Appendix 2 for full instrument). These questions examined metadata (Q2-4), the usefulness and relevance of retrieved content (Q1, Q5-8, Q15-16), latency and difficulty level of searches (Q9, Q12-14), IPR (Q11), and open sources (Q10).

Each question was answered on a Likert scale from 1 (strongly disagree) to 5 (strongly agree). The questionnaire, which was administered in an online format, was followed by two open-ended questions: (1) What are the positive and negative aspects of the system for searching and finding content?, and (2) What advantages and disadvantages do you perceive of a portal with international content?

## Results

This section presents the results of the evaluation of the metadata schema and the overall usability of the system, as well as how this was perceived and accepted by users. The section starts with the results of the administration of the SUS and of the questionnaire on the metadata and search process. Following, a subset of the questions from the questionnaire is mapped into the International Federation of Library Associations and Institutions (IFLA) Framework [41], for an aggregated analysis. We then present the results of the analysis of the two open-ended questions.

### Administration of the System Usability Scale

Table 1 presents the results of the evaluation using the SUS instrument from four different case studies that took place in four different countries (ie, Greece, Cyprus, Bulgaria, and Romania) between August 31, 2011, and April 7, 2012.

**Table 1:** SUS scores for the mEducator3.0 MELINA+ instantiation in four case studies.

Evaluation place	Evaluation as part of:	Group size/Profile	% SUS score for mEducator3.0/MELINA+
Plovdiv, Bulgaria	The e-Education and e-Science International Conference in Plovdiv October 5/6, 2011	25 medical professionals, students	56.6
Bucharest, Romania	An organized mEducator project event	15 medical students	62.8
Nicosia, Cyprus	An eHealth graduate class on November 11, 2011	35 postgraduate students taking an eHealth course	62.1
Thessaloniki, Greece	The Medical Education Informatics International Conference and Spring School on “Medical Education Content Sharing Technologies” on April 6/7, 2012	51 undergraduate medical students	64.7

**Figure 4:**
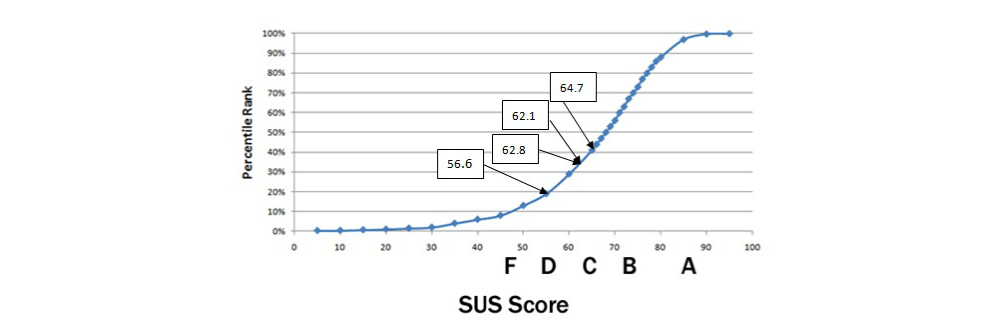
SUS scores of the four case studies of evaluation of mEducator 3.0 MELINA+ on the SUS curve.

Figure 4 shows how the SUS scores from the four case studies of the evaluation of MELINA+ are mapped on the SUS curve. Figure 4 shows that there is a progressive change of the overall system category from category D to C, over time, in the SUS score of the four case studies, starting from 56.6 in the first evaluation of MELINA+ in Bulgaria (category D) and reaching 64.7 in the fourth evaluation of mEducator MELINA+ in Greece (category C).

### Administration of the Questionnaire on the Metadata and Search Process

Results of this questionnaire constitute useful input for the analysis of the metadata accuracy to support the mEducator3.0 solution. It is important to note that the respondents’ level of expertise varied as it ranged from undergraduate students to medical professionals and professors (see Participants section). However, the results of the different groups (eg, students as opposed to professors or medical professionals) did not differ substantially to justify the presentation of results per group of users. Therefore, results are presented for all participants in Table 2.

**Table 2:** Results for the evaluation of the metadata and search process (N=126).

Category examined	Question	N	Strongly disagree, %	Disagree, %	Neutral, %	Agree, %	Strongly agree, %
1. Metadata	Q2. The metadata is not understandable	126	7.9	53.2	25.4	8.7	4.8
	Q3. The amount of presented metadata is excessive	124	1.6	39.5	38.7	16.9	3.2
	Q4. The amount of presented metadata is insufficient	124	5.6	48.4	33.9	12.1	0
2. Retrieved content usefulness and relevance	Q1. The presented metadata helps me in revising the search or annotation terms	125	0	7.2	24	61.6	7.2
	Q5. I found useful content as outcome of my searches	120	2.5	1.7	17.5	55.8	22.5
	Q6. The amount of retrieved relevant content was adequate to my information needs	115	0	7.0	32.2	52.2	8.7
	Q7. The information immediately presented helps me assess the relevance of the resource	118	0.8	9.3	32.2	51.7	5.9
	Q8. I need to inspect the learning resource to assess its relevance	116	0	12.1	33.6	47.4	6.9
	Q15. I found interesting content outside the scope of my specific search	114	2.6	8.8	28.1	46.5	14
	Q16. I would recommend the system to my colleagues	123	1.6	4.1	14.6	49.6	30.1
3. Latency and difficulty level of searches	Q9. The search results were obtained quickly	118	0.8	0.8	14.4	63.6	20.3
	Q12. The advanced search form is easy to understand	115	0.9	7.8	24.3	57.4	9.6
	Q13. It is distracting to have international content listed in the results	118	4.2	36.4	42.4	16.1	0.8
	Q14. It was easy to inspect/download the (retrieved) learning resource	123	2.4	8.9	30.1	46.3	12.2
4. Assessing open sources	Q10. I could easily assess if the resource is open to use	123	1.6	5.7	34.1	53.7	4.9
5. IPR	Q11. It was difficult to understand the IPR of the resources	123	0	34.1	41.5	20.3	4.1

For the part of the questionnaire examining *metadata* (three questions), data analysis showed that in general more than half of participants agreed or strongly agreed that the metadata were understandable (17/126, 61.1%) and that the amount of metadata was sufficient (67/124, 54%).

With regard to the second category, *the usefulness and relevance of retrieved content* (seven questions), more than half of participants agreed or strongly agreed that the content they retrieved as an outcome of their searches was useful (94/120, 78.3%), adequate for their information needs (70/115, 60.9%), relevant (68/118, 57.6%), interesting (69/114, 60.5%), and also helped them in revising their searches (86/125, 68.8%). It is also important to note that a greater percentage, almost 79.7% (98/123) of participants, would recommend the system to their colleagues.

The third category referred to the *latency and difficulty level of searches* (four questions). The majority of participants agreed or strongly agreed that the search results were obtained quickly (99/118, 83.9%), the advanced search form was easy to understand (77/115, 67%), and that it was easy to download the retrieved learning resource (72/123, 58.5%). Last, more than half of participants agreed or strongly agreed that they could easily assess whether the learning resource was *open to use* (72/123, 58.5%). It must be noted that this aspect of the system was explicitly included in the questions because mEducator needed an instrument capable of registering any difference in performance of the various instantiations, given the different technologies (mashups and semantic searches) that were underlying the two classes of mEducator solutions.

The participants’ response to a small number of questions was ambiguous and therefore difficult to interpret. What was problematic in the findings was that in some questions (Q3, Q4, Q7, Q8, Q10, Q11, Q13), typically one in three participants was neutral and did not express an opinion. For example, with regard to whether metadata was excessive (Question 3), more than one third of participants (48/124, 38.7%) were neutral, while one fifth of them (25/124, 20.1%) agreed with the statement. It is possible that in this case, participants were uncertain of what the concept of “excessive metadata” meant.

One important issue that needs to be understandable by the user is the IPR license that has been assigned to the resource. The results of Q11 (“It was difficult to understand the IPR of the resources”), although overall positive, also provide an indication of confusion among users with respect to the term IPR. The majority of participants either disagreed (42/123, 34.1%) or were neutral (51/123, 41.5%) with respect to this question. Neutral responses can be interpreted in a number of ways. They might mean that users are uncertain of their answer. If the results are analyzed from this point of view, then the implication is that the presentation of IPR metadata needs to be improved. Regarding the evaluation of the IPR schema of the repurposed content, it can be analyzed from two different perspectives. The first one refers to the case where the to-be-repurposed resource is already published in the system, and the second one refers to the case where the to-be-repurposed resource is an external one. According to the first one, the mEducator schema successfully takes care of the licensing of the to-be-repurposed resource because every resource that is already in the system has a license. However, regarding the second case, the IPR license of the external resource is currently implemented in mEducator platforms but not in the schema. More specifically, there is no IPR-related field in the schema about the repurposed resource when this is an external one to the system. This latter situation is handled by the platform by requiring the user to tick a box, thus giving assurance that the user is authorized to make use of the learning resource. However, this information is not saved in the metadata.

The next section presents a more aggregated analysis, which is based on groups of questions mapped into a general reference model for bibliographic metadata.

### Mapping the Online Survey into the International Federation of Library Associations and Institutions Framework: Aggregated Analysis

For convenience of interpretation, a subset of the questions from the online metadata accuracy survey was mapped into the IFLA Framework, namely the Functional Requirement for Bibliographic Record (FRBR) Framework [41]. The components of IFLA are (1) find, (2) identify, (3) select, and (4) obtain. Specific usability measures apply to these four generic tasks. Precision and helpfulness are important usability measures for the task of finding information. Ease of understanding, information adequacy, error rate, and information accuracy are important for both identifying and selecting information. Physical item accessibility is important for obtaining information. In this section, the responses to this subset of questions are analyzed, following the FRBR framework structure.

#### Find: Can a User Enter a Search and Retrieve Records Relevant to the Search?

By embedding the online metadata questionnaire into the IFLA Framework, the questions that were mapped under this stage of the IFLA framework are 5 and 6. Question 5 is “I found useful content as an outcome of my searches” and Question 6 is “The amount of retrieved relevant content was adequate to my information needs”.

**Figure 5:**
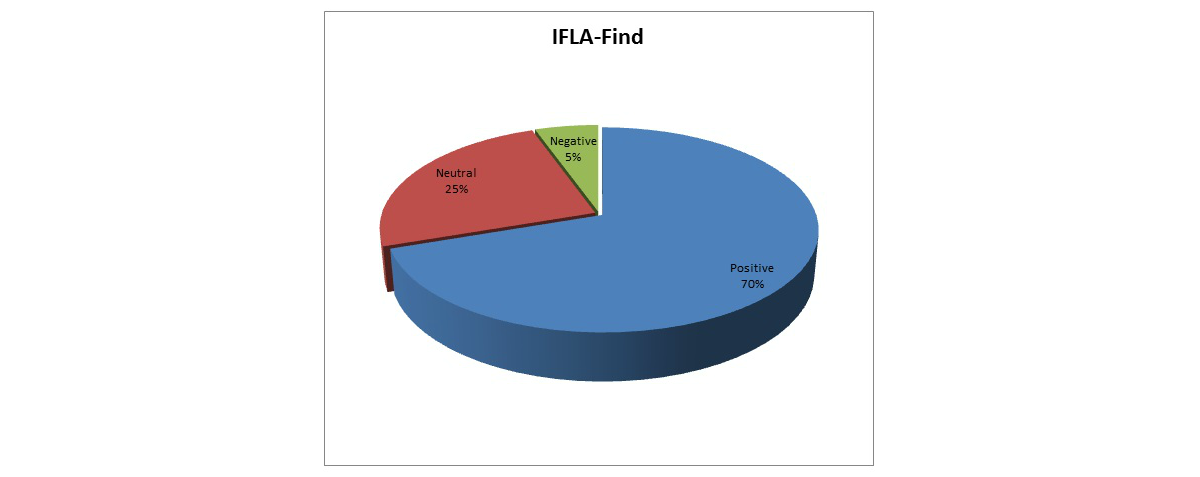
Users' responses mapped to the IFLA-Find requirement.

The results depicted in Figure 5 are based on a sample of 120 questionnaires and show whether a user can search and retrieve records relevant to the search. At first glance, it can easily be seen that 70.0% (84/120) of evaluators were satisfied regarding the retrieval of resources based on their needs. Not insignificant is the sum of neutral and negative results, which is 30.0% (25% +5%). Thus, approximately one third of evaluators (36/120) have either a negative or a neutral opinion on this.

Question 9, “The search results were obtained quickly”, is also related to this IFLA stage because it investigates the speed with which results are retrieved. The average value of Question 9 was 4.01 and shows a predominantly positive attitude toward speed of retrieval, suggesting that there were no unacceptable delays during the retrieval of resources.

#### Identify: Once the User Retrieves a Record, Can They Successfully Interpret the Information in the Record to Know Whether the Source Information Will Be Relevant to their Needs?

The results of this section are informative, as they show to what extent the metadata are presented in a user-friendly way to the end-users and if they make sense to them, so that they are able to assess the suitability of metadata to their needs. The questions of the online metadata survey that were analyzed at this stage are Question 1: “The presented metadata helps me in revising the search or annotation terms”, Question 2: “The metadata is not understandable”, Question 4: “The amount of presented metadata is insufficient”, Question 7: “The information immediately presented helps me assess the relevance of the resource”, Question 10: “I could easily assess if the resource is open to use”, and Question 11: “It was difficult to understand the IPR of the resources”.

**Figure 6:**
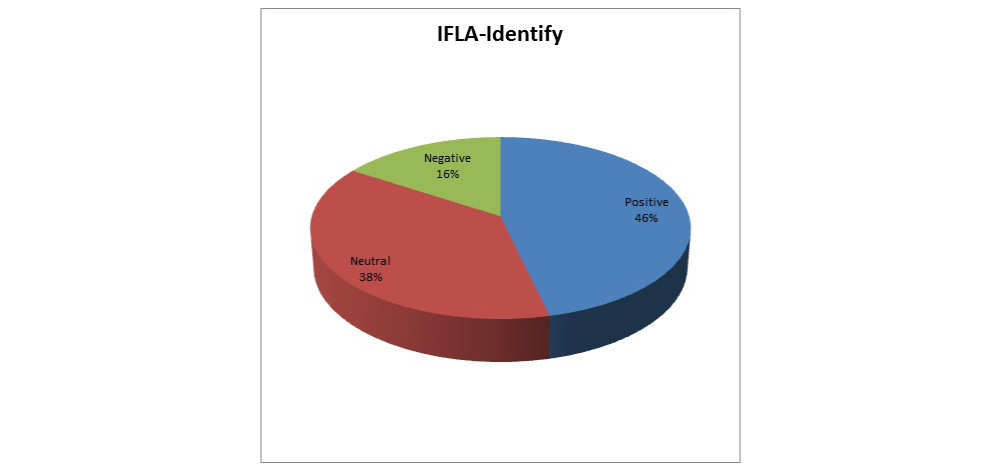
Users' responses mapped to the IFLA-Identify requirement.

Figure 6 shows that 45.8% (55/120) of participants agree that they can interpret the retrieved information and can understand if it meets their needs or not. The remaining 54.2% (65/120) express a neutral to negative attitude. These results are not satisfactory and indicate that the meaning of metadata is not sufficiently clear to the evaluators.

Independent from the analysis of the questions above (1, 2, 4, 7, 10, 11), another question from the online metadata accuracy survey that is related to the current stage of IFLA is Question 3. It is worth looking at the average value of Question 3 because this metric shows the length of the metadata, which indirectly affects the ability of the learner to interpret the metadata. More specifically, the average value of Question 3, “The amount of presented metadata is excessive”, is 2.8. That means that most people disagree that the amount of metadata is excessive. This is a positive remark because it encourages them to further investigate and look at the resource, so they can interpret it better.

#### Select: Can the User Compare the Information in Multiple Records and Determine the Most Relevant Record?

**Figure 7:**
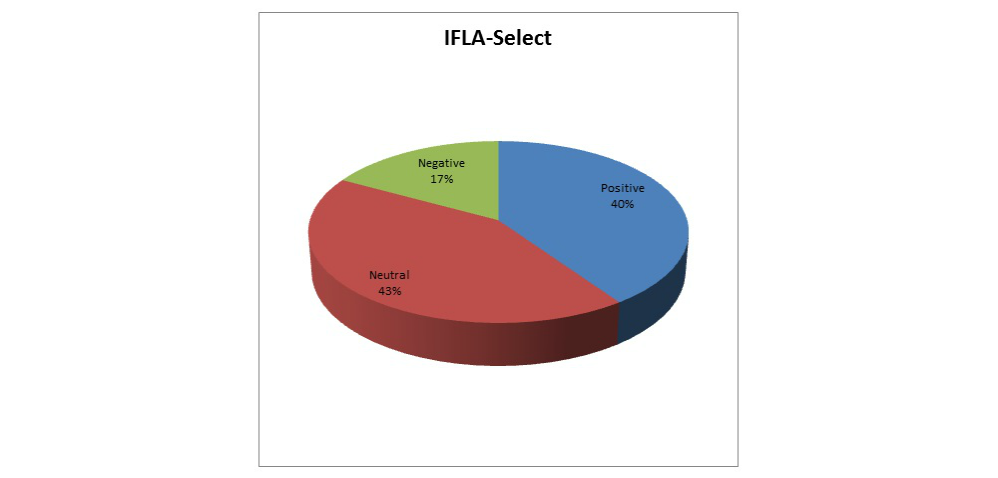
Users' responses mapped to the IFLA-Select requirement.

At this IFLA stage, whether the relevance of a resource can be easily determined from a list with multiple records is checked. Question 13, “It is distracting to have international content listed in the results”, addresses this issue. Responses to Question 13 are summarized in Figure 7.

The analysis was based on the multilinguality parameter. Figure 7 shows that 40.0% (48/120) of evaluators are positive, meaning that they disagree that it is distracting to have international content listed in the results. However, the greatest percentage of participants (51/120, 42.5%) falls into the “neutral” category, which suggests that they have either not understood the question or do not have an opinion. Additionally, 16.7% (20/120) are negative, meaning that they agree that the multilingual content is distracting. By combining the high percentage of neutral responses with the percentage of participants who agreed with the statement (24/120, 20.0%), we come up with a high percentage of evaluators (72/120, 60.0%) that cannot possibly determine the most relevant record among multiple ones.

#### Obtain: Can the User Successfully Obtain the Original Artefacts, Based on the Information Provided in the Source Information?

**Figure 8:**
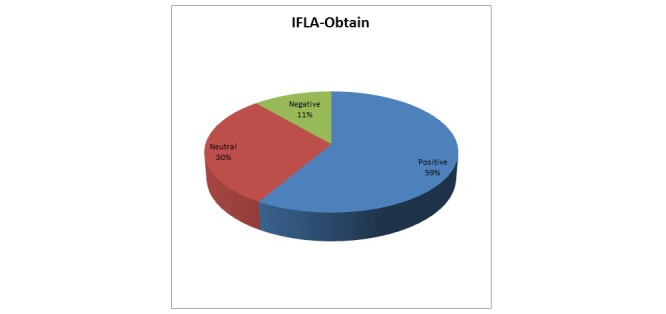
Users' responses mapped to the IFLA-Obtain requirement.

In order to ascertain users’ satisfaction regarding the accessibility of the original artifact, Question 14, which is “It was easy to inspect/download the retrieved learning resource”, was analyzed. Responses to Question 14 are summarized in Figure 8, which shows that 59.2% (71/120) of those questioned found it easy to download the retrieved learning resource. Although more than half of the respondents replied positively, the percentage of all the rest, 40.8% (49/120), is still important and should be considered.

### Users’ Input on the Strengths and Weaknesses of the mEducator Concept

The two open-ended questions proposed at the end of the survey aimed at eliciting an overall assessment from the user of the strengths and weaknesses of the mEducator concept mostly in relation to its overall usability and value. The two open-ended questions were (1) What are the positive and negative aspects of the system for searching and finding content?, and (2) What advantages and disadvantages do you perceive of a portal with multilingual content?

As a first step, the content of the participants’ answers to these two questions was analyzed for concept identification. As a second step, similar concepts with different wording were grouped into more comprehensive concepts, categorized as strengths and weaknesses. Each response was tested and re-tested for the associated concepts. Table 3 shows the details of these findings.

**Table 3:** Content analysis of open responses to the online survey indicating strengths and weaknesses of the mEducator system.

	Issue/Question	Count
Strengths	Awareness of international standards in digital medical education	12
	Overall, easy to use even without specific knowledge	12
	Linking with colleagues, fostering collaboration, peer reviewing	11
	Varied, interesting, up-to date content, from different providers	11
	Supports evaluation of scientific topic from different perspectives	5
	Easy access, worldwide	3
	Narrow down concepts through advanced search	3
	Can publish my own content, contribute/search in any language	3
	Specificity of information, as opposed to other search platforms	2
	Usefulness of subject profile for finding content	2
	Good concept	2
	Detailed info on terminology	1
	Is in English	1
Weaknesses	Slow search	14
	Need for translation	11
	Not user-friendly, difficult to use at the beginning	9
	Technical jargon, specific knowledge required	7
	Little variety, not enough, or irrelevant content	7
	Complex search form, issues with filtering	6
	Navigational/presentation issues	6
	Difficult to understand all content, when in foreign language	5
	Distributed semantic search difficult to understand	4
	Some items could not be accessed	3
	Some bugs	3
	No language support	3
	Most articles in English	2

The open responses provided an interesting overview of the aspects that the users spontaneously highlighted as most valuable to them. Among the strengths, the opportunities to be aware of multilingual perspectives and activities on the same topic, such as peer collaboration for research and publications purposes, were highlighted as important by users. Less frequently mentioned was the opportunity to publish own materials and the specificity of the content in the platform. Among the perceived weaknesses, recurrent themes were the slowness of the distributed search, the technical terminology that was difficult for users to understand, and the need for translation. Users’ suggestions for the improvement of the mEducator system included developing a special edition of the mEducator platform to be accessed only by students, keeping the interface as simple as possible (eg, like Google), protecting against irrelevant data, providing more flexible filters for languages, and prioritizing search parameters.

## Discussion

### Principal Findings

This study has described how a scenario-based evaluation framework has been applied to one instantiation of the mEducator BPN (ie, mEducator3.0, MELINA+) for searching and sharing medical educational content and has presented the results of the assessment of the metadata schema used in the system and its usability and acceptance by users. The main findings of the study are that (1) the informational needs of the mEducator target groups were addressed to a satisfactory extent with regard to the process of medical content searching and sharing, (2) the metadata schema supported searching for, retrieving, and sharing of content from an end-user perspective, and (3) the overall usability of the mEducator3.0 MELINA+ system was acceptable based on the results of the SUS scores from 126 participants. Also, the study pointed out that, among the various possibilities offered by the mEducator approach to content sharing and repurposing, target users valued being aware of international standards and being able to benchmark the resources used in other educational programs, and also being able to link with resources of their peers. In accordance with previous research [22-24], this study adopted a user-centered approach. However, one important aspect that differentiates this study from previous studies is that in the case of mEducator, completion of metadata is not performed by professional indexers [16] but is largely delegated to end-users and content providers. This is challenging because of difficulties in achieving a shared understanding of the meaning of the metadata fields. The results of this study confirmed that the meaning of metadata was not sufficiently clear to some evaluators. As reported in the facilitator’s and research assistants’ field notes, some specific examples where the meaning of metadata was unclear refer to descriptions of technical terms, such as “identifiers”, “quality stamp”, “URI”, “URL”, “ISSN”, “ISBN”, “platform users”, and “external users”, to name a few. Moreover, a rather high percentage of evaluators could not determine the most relevant record among multiple ones, and this could have been a result of the multilingual parameter of the system. Finally, another weakness that was documented referred to users’ lack of knowledge about IPR issues, which are very important in repurposing content in medical education.

In the next section, implications for the evaluation of the processes of searching, retrieving, and sharing of data are drawn by addressing issues of content description metadata, content description procedures, and IPR for repurposed content, in the form of recommendations and lessons learned.

### Recommendations and Lessons Learned From the Scenario-Based Evaluation

The scenario-based evaluation provided us with a broad and contextualized view on the user experience related to mEducator services. Repurposing and re-use of medical educational resources is becoming vital in the current economy as many of these resources are costly to produce. Systems for locating suitable medical or other content need to be improved to allow this to happen, therefore recommendations in this respect are proposed in this paper. Even though in this study, (1) medical-domain specific metadata were defined and used, (2) medical-domain–related scenarios were constructed and used, and (3) all participants were related with the medical domain, these recommendations not only apply to systems of medical education whose aim is to facilitate sharing of medical content but also apply to different systems of searching for and sharing content.

Designers of systems of searching for, retrieving, and sharing medical content should:

- emphasize ease of use, by referring to the most current best practices of usability and benchmarking their service with widely used tools (eg, Google or YouTube) and their prevailing conventions of use while simultaneously acknowledging the need for functionalities validating scientific content
- aim for smooth and effective use, by making sure the technology used does not interrupt or slow down the workflow or for example, the progressive search process
- provide support for finding and retrieving content by introducing an extensive schema in a prioritized order with the most critical metadata in front
- provide support for indexing content, that is, creating high-quality metadata, by using for example, suggestions and hints as well as dynamic, responsive annotation forms when dealing with an extensive, complex schema
- base the user interaction on the expected concepts that are familiar to the user, by simplifying the novel terminology and/or using a vocabulary with clear descriptions of technical terms (such as IPR, identifiers, quality stamp, URI, URL, ISSN, ISBN)
- support multilinguality in terms of the user experience as well as of content
- highlight the advantages, that is, the added value from using the system, for example, by offering explicit information on the type of multilingual content and system functionality available
- allow inputting metadata in various formats, especially regarding licenses or identifiers that are not compliant with users’ content or are not familiar to them
- support community creation, that is, sharing contacts and content with other medical professionals, students, general practitioners, etc (this is a characteristic of mEducator and is a feature not offered by similar systems)

Based on the findings of this study, we can make several recommendations on how to improve the search, retrieval, identification, and gathering of medical resources based on the provided metadata. These recommendations not only relate to the mEducator metadata but apply to any platform that processes medical or other types of metadata to retrieve educational resources. The first recommendation is to include the option of an “advanced search”. This is expected to facilitate managing retrieved results. The second recommendation is to allow different orderings of the retrieved results, for example, based on type of resource or author name. This is expected to facilitate the identification of relevant resources from among the potentially huge amount of retrieved results/records. A third recommendation is to list search terms under the results. An example would be the display of a message such as “Your search for this keyword resulted in x number of results”. This feedback will enable the user to have a general idea of the quantity of retrieved results for a specific keyword or combination of keywords. Last, assessing the relevance of a resource to the user’s needs before inspection can be improved in a number of different ways. One of these ways refers to the use of icons next to the list of results or also in the metadata page that could inform the users before they view the resource, that it is an “open resource” (ie, easily/freely accessible) or a resource that requires a login. Another way refers to the use of a “dashboard” in the results page, to display useful information, such as how many times the resource has been downloaded. Last, the interpretation of metadata can be improved by a thumbnail of the actual resource that should be made available while viewing the metadata.

With respect to IPR clearance, the most sensible recommendation is to devote some effort to educating the user about the options available through creative commons licensing and easing the process of obtaining it, for example through the implementation of a step-wise process to guide the user in obtaining IPR clearance.

The users of the mEducator who want to share their content will have to specify how other users will be entitled to use it. Some recommendations derived from the evaluation efforts are the following: a better explanation of the meaning of the IPR abbreviation and options should be provided, an automated mechanism should be provided to check the validity of the IPR and prevent the user from providing any resource’s link as long as the IPR had not been provided, and a policy should also be defined on how to deal with contents from external repositories for which the IPR clearance is unknown.

With regard to how the system has been adopted by the e-learning and medical community since 2011-2012 when the evaluations reported in this paper took place, it is important to note that one of the main objectives of the mEducator project was to establish standards. To this extent, the strategic decision of the consortium was to link and be involved with the activities of the Medbiquitous Consortium, an Organization producing standards for digital health education. Several fields of the mEducator schema have already been incorporated as extensions in the current process of revision of the Healthcare Learning Object Metadata (LOM) standard towards version 2.0, by the Technical Committees and LOM Working Group of Medbiquitous. Finally, it is important to mention that the mEducator3.0 MELINA+ system is under routine use in some partner organizations, but it has also been installed and trialed in other associate partners, health organizations, and IT facilities. For example, the Institute for the Study of Urological diseases is one of those using the mEducator Melina+ system.

### Limitations

There were several limitations in this study, specifically, the accuracy of metadata in the stricter sense. We did not address if the metadata actually describes the educational resource that it refers to. The limited time that participants could voluntarily devote to the evaluation of mEducator as part of the different workshops, conferences, and evaluation events that were organized prohibited the use of controlled, experimental usability conditions and allowed for the administration of only two evaluation instruments and the documentation of users’ difficulties through field notes. The first one of these evaluation instruments, the SUS, although generic, is a standardized instrument that was used to evaluate the mEducator3.0 MELINA+ overall usability in absolute terms, so that it can be comparable to other instantiations of the system. The second instrument was customized to address the needs of the study. Moreover, field notes taken during the evaluation sessions were used as an additional data source for triangulation purposes.

It is important to note that additional evaluation methods have been implemented in the scope of the overall study, including interviews, observational case studies, screenshot capturing, and automated tracking of activity in the different evaluation events organized by mEducator. However, these evaluation events provided space for informing initial designs and obtaining user preferences and user attitudes. The results from these different data sources were mainly used to inform the first prototype of the mEducator system in the initial stage of the project and are not reported in this study. However, final evaluations reported in this study were aiming towards more specific and important goals such as the elicitation of recommendations to the medical community.

### Conclusion

The mEducator solutions (mEducator2.0 and mEducator3.0) are part of a family of tools. These tools aim to gather structured data according to the same metadata schema. The functionalities of these tools allow users maximum control over the search process, access to detailed but understandable results in order to know very quickly if the retrieved information is linked to content that is accessible or not, and the ability to browse the information in an efficient manner even if they are not entirely sure what they are looking for.

When using the mEducator services, the users’ main objective is to find the resources they need in the most efficient manner. Facilitating and guiding query formulation is one of the major objectives of the mEducator tools. It is important to note that while most general purpose Web search engines deal mostly with unstructured data (and a small amount of structured data), mEducator contains almost exclusively data compliant with the mEducator metadata schema, which greatly simplifies the process of guidance in query formulation.

The different instantiations of mEducator offer somewhat different search options. Where mEducator2.0 searches only for content, the mEducator3.0 systems also allow searching for members, and some of them (eg, MetaMorphosis+ and MELINA+) allow searching for more detailed types of content such as exercises, simulations, presentations, or virtual patients. They even allow some control over the presentation or sorting of results. An important option the systems share is the option to select resource language.

The goal of this study was to implement a scenario-based evaluation methodology framework of one instantiation of the mEducator BPN (mEducator3.0/MELINA+) to assess both the metadata schema used as well as the system’s overall usability and acceptance by users. The study defined the extent to which the informational needs of the mEducator target users were covered and demonstrated that the metadata schema implemented in the MELINA+ instantiation of the mEducator3.0 solution supported the searching, retrieval, and sharing of content from an end-user perspective. The study is original from a methodological point of view in that it implemented scenario-based assessment for evaluating content-sharing technologies including the interactions between end-users, content, and technology. The recommendations and lessons learned (derived with respect to content description metadata, content description procedures, and IPRs for repurposed content), the whole framework, and the results of the evaluation impact researchers, medical professionals, or other groups interested in using similar systems for educational content sharing in other domains.

## Appendix

Multimedia Appendix 1 SUS questionnaire.

Multimedia Appendix 2 Questionnaire on the metadata and search process.

## Abbreviations

BPN: best practices network
CMS: content management system
CPR: cardiopulmonary resuscitation
FRBR: Functional Requirement for Bibliographic Record
HCI: human-computer interaction
LOM: learning object metadata
IFLA: International Federation of Library Associations and Institutions
IPR: intellectual property rights
ISBN: international standard book number
ISSN: international standard serial number
LCMS: learning content management system
MELINA: MEdical Education LINnked Arena
MELT: A Metadata Ecology for Learning and Teaching
SUS: System Usability Scale
URI: uniform resource identifier
URL: uniform resource locator
