# Supplementary material for: Medical Content Searching, Retrieving, and Sharing Over the Internet: Lessons Learned From the mEducator Through a Scenario-Based Evaluation
Source: J Med Internet Res. 2015 Oct 9;17(10):e229. doi: 10.2196/jmir.3650 (PMC4642372; doi:10.2196/jmir.3650)
Supplement: Multimedia Appendix 1 [file jmir_v17i10e229_app1.pdf]

APPENDIX 1

SUS questionnaire

The following is the 10-question SUS questionnaire.

1. I think that I would like to use this system frequently.
2. I found the system unnecessarily complex.
3. I thought the system was easy to use.
4. I think that I would need the support of a technical person to be able to use this system.
5. I found the various functions in this system well integrated.
6. I thought there was too much inconsistency in this system.
7. I would imagine that most people would learn to use this system very quickly.
8. I found the system very cumbersome to use.
9. I felt very confident using the system.
10. I needed to learn a lot of things before I could get going with this system.
