# Supplementary material for: Medical Content Searching, Retrieving, and Sharing Over the Internet: Lessons Learned From the mEducator Through a Scenario-Based Evaluation
Source: J Med Internet Res. 2015 Oct 9;17(10):e229. doi: 10.2196/jmir.3650 (PMC4642372; doi:10.2196/jmir.3650)
Supplement: Multimedia Appendix 2 [file jmir_v17i10e229_app2.pdf]

APPENDIX 2

Questionnaire on the metadata and search process

1. The presented metadata helps me in revising the search or annotation terms.
2. The metadata is not understandable.
3. The amount of presented metadata is excessive.
4. The amount of presented metadata is insufficient.
5. I found useful content as outcome of my searches.
6. The amount of retrieved relevant content was adequate to my information needs.
7. The information immediately presented helps me assess the relevance of the resource.
8. I need to inspect the learning resource to assess its relevance.
9. The search results were obtained quickly.
10. I could easily assess if the resource is open to use.
11. It was difficult to understand the IPR of the resources.
12. The advanced search form is easy to understand.
13. It is distracting to have international content listed in the results.
14. It was easy to inspect/download the (retrieved) learning resource.
15. I found interesting content outside the scope of my specific search.
16. I would recommend the system to my colleagues.
